# Supplementary material for: Clinical Evaluation of Metagenomic Next-Generation Sequencing Method for the Diagnosis of Suspected Ascitic Infection in Patients with Liver Cirrhosis in a Clinical Laboratory
Source: Microbiol Spectr. 2023 Jan 10;11(1):e02946-22. doi: 10.1128/spectrum.02946-22 (PMC9927505; doi:10.1128/spectrum.02946-22)
Supplement: Supplemental file 2 — Supplemental material. Download spectrum.02946-22-s0002.pdf, PDF file, 0.7 MB [file spectrum.02946-22-s0002.pdf]

Clinical evaluation of metagenomic next-generation sequencing method for the diagnosis of suspected ascitic infection in patients with liver cirrhosis in a clinical laboratory

Running title: mNGS method for ascitic infection diagnosis

Hao-Xin Wu [1†], Fei-Li Wei [2†], Wei Zhang [1], Jie Han[1], Shan Guo[2], Zheng Wang [1], De-Xi Chen[2], Wei Hou[1*],Zhong-Jie Hu [1*]

[†]Author Hao-Xin Wu and author Fei-Li Wei (co-first authors) contributed equally to this paper.

[*]Author Zhong-Jie Hu and author Hou Wei are the co-corresponding authors.

[1] Beijing Youan Hospital, Capital Medical University, Beijing, China.

[2] Beijing Institute of Hepatology, Beijing YouAn Hospital, Capital Medical University, Beijing Precision Medicine and Transformation Engineering Technology Research Center of Hepatitis and Liver Cancer , Beijing, China.

***Corresponding author**: Dr. Zhong-Jie Hu

Address: No.8, West Tou Tiao Community, Youan Men Wai Street, Fengtai District, Beijing

Telephone number: 13501366613

Email: huzhongjie@ccmu.edu.cn.

Dr. Wei Hou

Address: No.8, West Tou Tiao Community, Youan Men Wai Street, Fengtai District, Beijing

Telephone number: 13671279263

Email: baoerlanglang@163.com

Keywords: Metagenomic next-generation sequencing, ascitic infection, diagnosis, clinical laboratories

Supplemental materials:

Diagnostic gold standard. Whenever patients develop any of the following: 1) acute peritonitis: abdominal pain, abdominal tenderness or rebound tenderness, an increase in abdominal muscular tension, vomiting, and diarrhea or intestinal obstruction; 2) systemic inflammatory response syndrome: fever or normothermia, shivering, tachycardia, and tachypnea; 3) deterioration of liver function due to an unknown underlying cause; 4)hepatic encephalopathy; 5) shock; 6) refractory ascites, no response to diuretics, or renal failure; 7) acute gastrointestinal tract bleeding. And one or more of the following laboratory test abnormalities are present: 1) polymorphonuclear (PMN) ≥250 cells/mm3; 2) positive ascites bacteria culture; 3) procalcitonin (PCT) >0.5 ng/mL, and infection of other sites is excluded. (EASL guidelines, J Hepatol 2018; Chinese guidelines, Hepatol Int 2019; AASLD Practice guidance, Hepatology 2021).

Inclusion criteria. Inclusion criteria were age greater than 18 years and cirrhosis diagnosed by either liver biopsy or a combination of clinical, analytic, ultrasound, elastographic, and/or endoscopic findings. Screening patients with cirrhosis (no etiology limitation) complicated with spontaneous bacterial peritonitis, bacterascites and ascites without infection.

Exclusion criteria: acute HAV ,HEV ,EBV or CMV virus infection or infection of other sites. Ascites caused by other reasons, such as renal ascites, cardiac ascites, pregnancy drug abuse or HIV infection. Uncontrolled liver cancer or other systemic tumors. Previous organ transplant recipients receiving glucocorticoids or other

immunosuppressive therapy. Rifaximin used in the past one month.

Supplemental methods

Blood culture and pathogen identification

We processed the samples in the microbiology laboratory as follows: Ascitic fluids obtained from each patient were injected into automatic blood culture bottles (Aerobic, Anaerobic and Myco/F Lytic bottles, 8–10 mL per bottle) to culture aerobic bacteria, anaerobic bacteria, mycobacteria, and fungus in automated blood culture system (BD BACTEC FX and BD BACTECTM 9120). When the system showed a positive growing signal, the sample was extracted from the bottle for Gram staining or Acid-fast staining to make smear microscopy, followed by subculture on Columbia blood agar and Maconkey Aga plate at 37°C with 5% CO2 for aerobic bacteria or without oxygen for anaerobic bacteria. If the system showed a positive blood culture signal for Myco/F Lytic bottle, besides smear microscopy, the sample was extracted from the bottle and cultured on SDA agar for fugus at 28°C. The pathogens cultured on the agar were further identified by matrix-assisted laser desorption-ionization time-of-flight mass spectrometry (MALDI-TOF MS; VITEK MS system, bioMérieux, France). The negative result without positive growing signal was reported when the samples were cultured 5 days for aerobic and anaerobic bacteria, 14 days for fungi, 42 days for mycobacteria in the automatic blood culture system.

Digital droplet PCR assay.

Sample processing.

1) To deplete the extracellular DNA from the ascites samples, 1 ml samples were centrifuged at 4°C with 13000r for 10 min.

2) After discarded 760 µl supernatant, we added 40.5 µl mixture of buffer and benzonase endonuclease (Sigma) and incubated this mixture at 37 °C for 15 min.

3) Next, we added 20 µl of protease K (Qiagen) and incubated it at 56°C for 20 min to inactivate the Benzonase.

DNA extraction.

4) 400 µl 2 × DNA / RNA shield (Zymo) was added into each of the pre-treatment samples.

5) After mixing, the samples were homogenized using a program of 4 °C 60Hz for 120 seconds, stopped for 20 seconds, repeated for 4 times in Tissuelyser (Servicebio), and then centrifuged at 10000r for 2 min.

6) We then individually added 400 µl DNA/RNA analysis buffer, prep buffer and wash buffer for repeated DNA washing. Finally, 50 µl DNase/RNase-free water was added to collect target DNA and stored at - 80°C until tested.

Droplet preparation and detection.

7) ddPCR was performed with the TargetingOne Digital PCR System (TargetingOne). The master mix for ddPCR included 1× ddPCR supermix for probes, 400 nmol/L of forward and reverse primers, and 200 nmol/L GRAM+/GRAM- probes;

8) 1 µl sample of DNA and DNase/RNase Free water were mixed together, and the final volume was 30 µl for each well. The droplet was generated according to the manufacturers' protocols.

9) PCR amplification was performed with the following conditions: 95°C for 10 min followed by 40 cycles of denaturation at 95°C for 10 min; annealing and extension

was at 60℃ for 1 min.

10) The strip tubes were stored at 4℃ until the droplets were analyzed with a TargetingOne chip reader and TargetingOne ddPCR Analyzer 1.0. The threshold between positive and negative droplet populations was manually set using per-plate positive and no-template controls as a guide.

The cutoff value of the bactDNA quantification was 103.2 copies/μl compared to the SBP diagnosis. For patients infected with Gram-positive and Gram-negative bacteria, the cutoff values were 37.3 and 68.6 copies/μl, respectively.

Prospective case series of mNGS testing in patients with suspected infection

Case 1

A 55-year-old man with liver cirrhosis attributed to alcohol (60g per day for 20 years) was hospitalized with a history abdominal distension despite oral diuretic treatment. The patient had Child-Pugh grade-C at admission (MELD score of 13). He showed signs of systemic inflammation manifested by pyrexia and increased PCT and C-reactive protein. Blood cultures and diagnostic paracentesis were obtained and empirical treatment with Piperacillin/tazobactam was initiated. Diagnostic paracentesis did not indicate SBP, with total white-cell count and PMN of 198 cell/mm^3 and 102 cell/mm^3, respectively. Two days later, the mNGS test identified *Enterococcus faecalis* and *Candida albicans* from ascites. The *E. faecalis* was confirmed by conventional cultures (3 days later), whereas the *C. albicans* was not be found. Additional testing including the sputum culture, midstream urine culture and fecal culture were negative. Five days later, the total white-cell count and PMN increased to 408 cell/mm^3 and 192 cell/mm^3, respectively. The Fluconazol was add to 7-day course (400mg, qd), and the piperacillin tazobactam was continued for *E. faecalis*. Repeated mNGS and conventional cultures of ascites were negative after antimicrobial therapy. He was discharged without symptoms of infection.

Case 2

A 45-year-old man with HBV-related cirrhosis was hospitalized for upper gastrointestinal bleeding and abdominal distension. He was receiving Entecavir for 5

years. He had Child-Pugh grade C (MELD score of 12) at admission. The findings from ascitic fluid showed the total white-cell count and PMN were 170 cell/mm^3 and 27 cell/mm^3, respectively, which were not consistent with the diagnosis of SBP. He was treated empirically with Cefotaxime sodium/sulbactam. Two days later, the mNGS testing identified *Enterococcus faecium*. The patient developed fever (38.0°C) on the third day, and the repeated diagnostic paracentesis indicated an increased ascitic fluid white-cell count and PMN (223 cells/mm^3 and 106 cells/mm^3), while the ascitic cultures were negative, and Teicoplanin was added. Seven days later, ascitic mNGS and conventional cultures were negative after antimicrobial therapy. He was discharged without symptoms of infection.

Case 3

A 53-year-old man with HBV/HCV-related cirrhosis and liver cancer was hospitalized with abdominal pain and at admission had Child-Pugh grade-B (MELD score of 12). The patient was diagnosed as SBP with ascitic white-cell of 9273 cell/mm^3 (PMN of 8000 cell/mm^3) and increased PCT and C-reactive protein. He was treated empirically with Vancomycin and Imipenem. The mNGS detected *Enterobacter cloacae*, *Enterobacter hormaechei*, *Escherichia coli*, *Stenotrophomonas maltophilia*, *Klebsiella pneumoniae*, whereas culture only identified *S. maltophilia*. Three days later, WBC, PCT, and ascitic PMN decreased and culture showed *E. cloacae*. Imipenem was discontinued and piperacillin was added to continue antibiotic treatment for one week. The patient had no fever or concomitant symptoms and was allowed to leave the hospital.

Case 4

A 68-year-old male with HBV-related liver cirrhosis and liver cancer presented with a 3-day interval fever with a maximum temperature of 39.5°C. He hospitalized with abdominal pain and skin and sclera icteric in emergency room. The result showed that ascitic white-cell was 210 cell/mm^3 (PMN 88 cell/mm^3) and culture identified *Enterococcus faecium*. He was treated with Imipenem and Tigecycline. The patient had obvious abdominal pain and tenderness after two days and the ascitic PMN increased to 1188 cell/mm^3. The mNGS detected *Klebsiella pneumoniae*, *Enterococcus faecalis* and *Enterococcus faecium*. Then Tigecycline was discontinued and Linezolid was added to continue antibiotic treatment. The result of culture was obtained 2 days later.

Case 5

A 58-year-old man with alcoholic-related cirrhosis was hospitalized with abdominal pain and tenderness and pyrexia of 38℃, and at admission had Child-Pugh grade-C (MELD score of 17). Ascitic white-cell was 532 cell/mm^3 (PMN 122 cell/mm^3), which was suspected with SBP infection. The mNGS identified *Enterococcus faecalis* at the second day, whereas culture was negative. Imipenem was empirically used for anti-infection. He was discharged without symptoms of infection.

Case 6

A 66-year-old woman with primary biliary cirrhosis was hospitalized with upper gastrointestinal bleeding and hepatic encephalopathy and at admission had Child-Pugh grade-B (MELD score of 19). The ascitic fluid was sent for culture and mNGS testing in the first and fourth day. *Escherichia coli* was confirmed by mNGS testing within 24h

but by culture with 5 days. She was treated with Latamoxef antibiotic treatment. And then, the second results showed that mNGS identified *Escherichia coli*, whereas culture was negative. Latamoxef was continued for one week and the ascitic fluid decreased from 368 to 202 cell/mm^3 (PMN from 137 to 44 cell/mm^3). She was discharged with a better health condition.

Case 7

A 63-year-old woman presented with cirrhosis of unknown cause, acute-on-chronic liver failure, and upper gastrointestinal bleeding. She was at admission with Child-Pugh grade-C (MELD score of 28), increased PCT, and ascitic white-cell of 49 cell/mm^3 (PMN of 13 cell/mm^3), which was suspected with SBP infection. The organism of *Escherichia coli* was identified by mNGS and Biapenem was used for antibiotic treatment. He was discharged without symptoms of infection after one week.

Case 8

A 54-year-old man with HBV-related cirrhosis and acute-on-chronic liver failure was hospitalized with abdominal pain and tenderness, increased PCT. The results from ascitic fluid showed the total white-cell count and PMN were 540 cell/mm^3 and 128 cell/mm^3, respectively, which were not consistent with the diagnosis of SBP. Blood cultures and diagnostic paracentesis were obtained and empirical treatment with Imipenem was initiated. The organism was confirmed by mNGS as *Enterococcus faecalis* after 2 days. Vancomycin was added to continue antibiotic treatment for one week. The patient’s health condition was improved.

Supplemental Figures

Supplementary Figure 1. The turnaround time of mNGS testing.

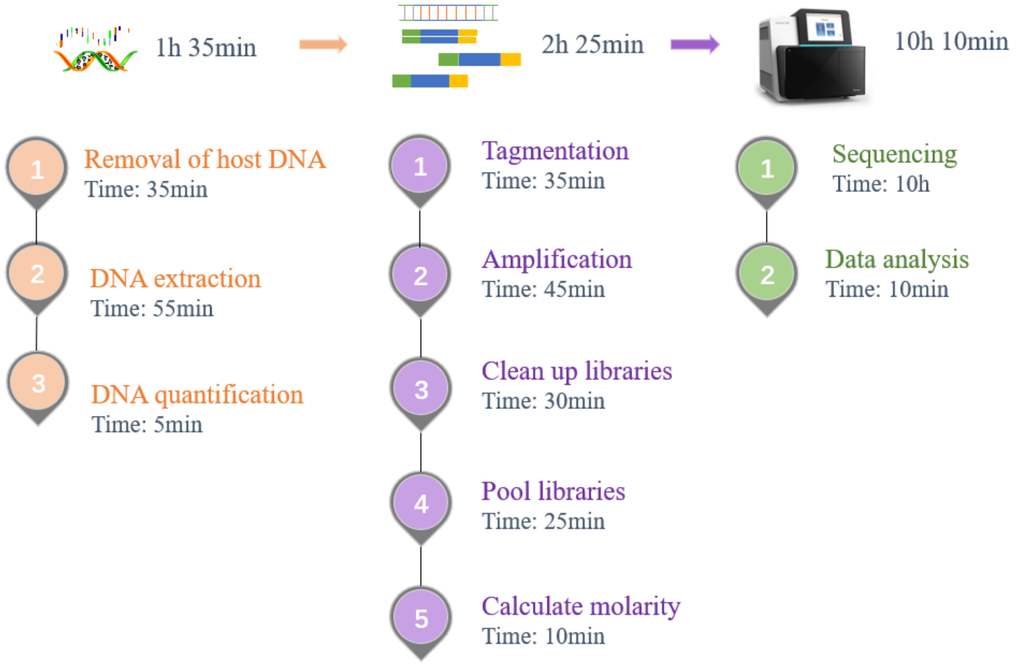

Supplemental Figure 2. Relationship of control positive organisms titer with the mNGS detection signals (normalized reads per ten million[nRPTM]).

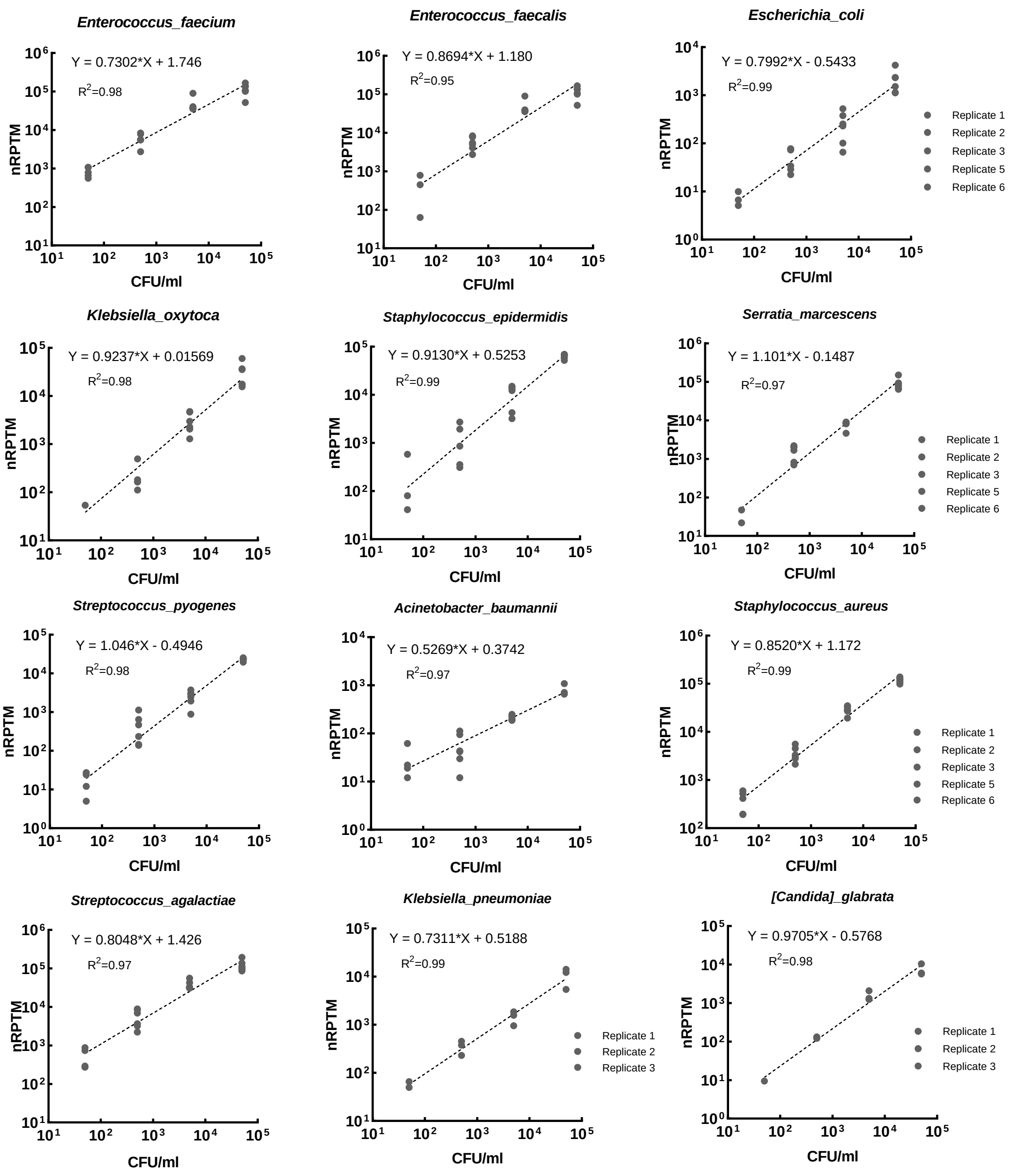

Supplemental Figure 3. Accuracy of mNGS testing.

A.

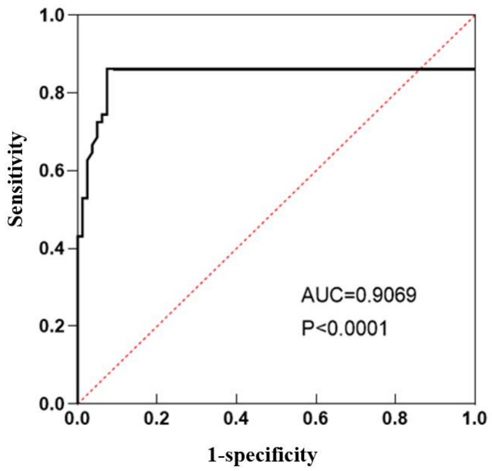

B.

conventional clinical testing		mNGS +	mNGS -
	+	32	6
	-	18	82

Sensitivity = 84.2%
Specificity = 82.0%
PPA = 64.0%
NPA = 93.2%

conventional clinical testing + Digital PCR + clinical adjudication		mNGS +	mNGS -
	+	41	6
	-	9	82

Sensitivity = 87.2%
Specificity = 90.1%
PPA = 82.0%
NPA = 93.2%

(A) ROC curves of normalized reads per ten million (nRPTM) from 67 samples based on clinical composite standards; **(B)** The 2×2 contingency tables for the validation of the nRPTM based on a composite standard.

ROC: receiver operating characteristic; PPA: Positive predictive agreement; NPA: negative predictive agreement;

Supplemental Figure 4. Characteristics of peritoneal infection in SBP patients.

A

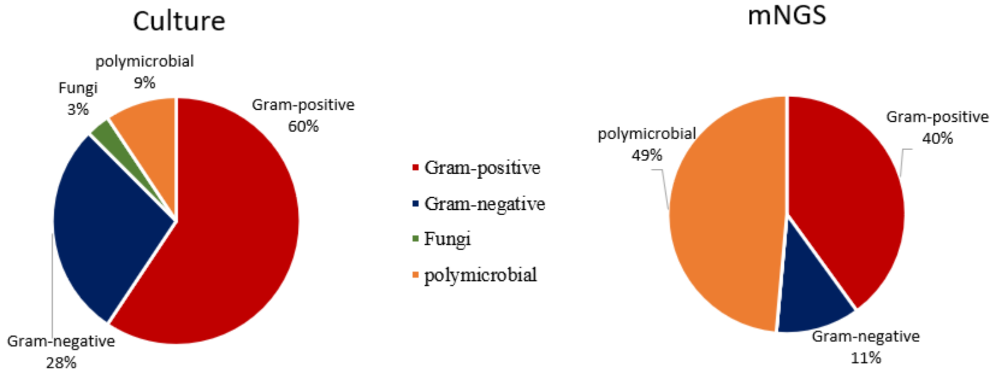

B

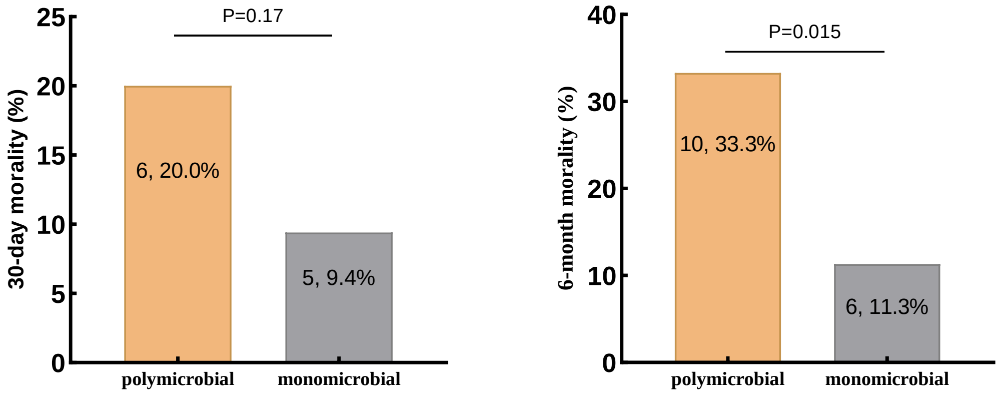

(A) Comparison of culture and mNGS identification in terms of pathogen species in SBP patients; **(B)** Comparison of monomicrobial and polymicrobial infection in terms of 30-day and 6-month morality.

SBP: spontaneous bacterial peritonitis.

Supplemental Figure 5. Comparable analysis in diagnosing SBP of three detection methods

A

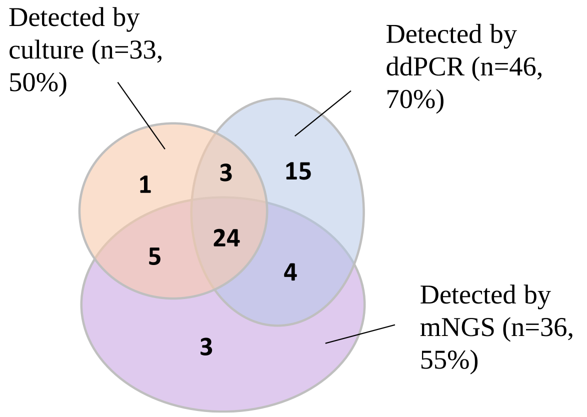

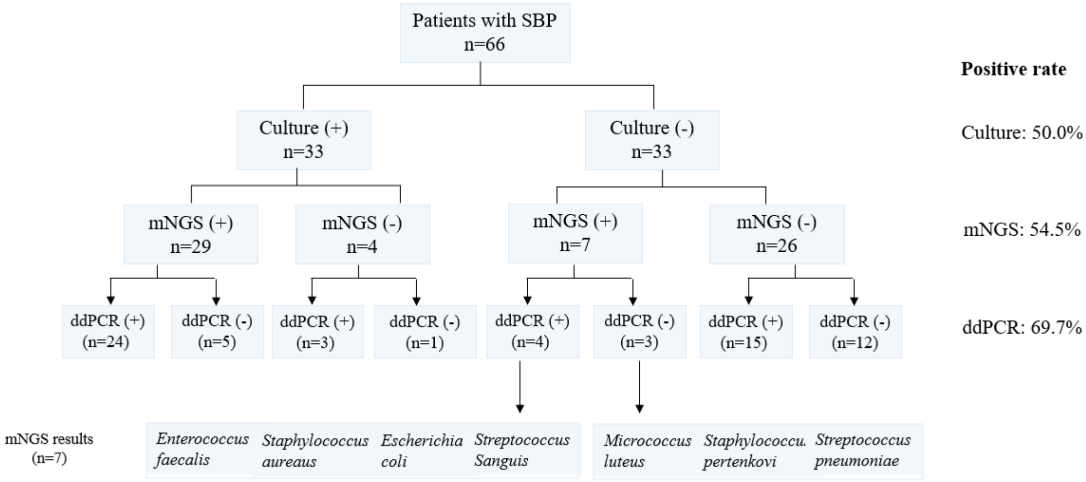

B

		culture pos	culture neg	mNGS pos	mNGS neg	ddPCR pos	ddPCR neg
SBP	pos	33	33	36	30	46	20
	neg	39	100	45	94	18	121

culture	mNGS	ddPCR
Sensitivity = 50.0%	*Sensitivity* = 54.5%	*Sensitivity* = 69.7%
Specificity =71.9%	*Specificity* =67.6%	*Specificity* =87.1%

C

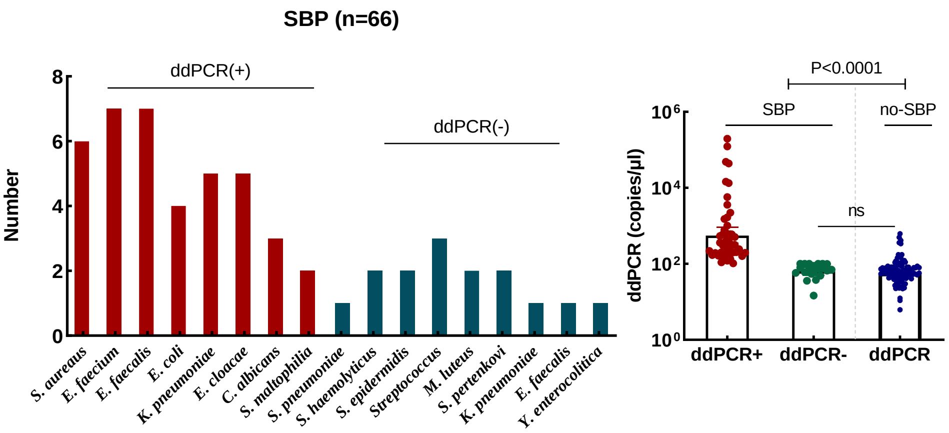

(A) The positive rate in the diagnosis of SBP according to culture, ddPCR, and mNGS, respectively; **(B)** Analytical sensitivity and specificity data comparing culture, ddPCR, and mNGS. **(C)** Comparison of ddPCR-positive and ddPCR-negative identification in terms of pathogen species in SBP patients.

SBP: spontaneous bacterial peritonitis; ddPCR: digital droplet PCR.

Supplemental Tables

Supplemental Table 1. 12 common strains of peritoneal infection.

Cat No.*	Organism Name	Class Type	Original Concentration (CFU/mL)	Strain Number
CA0741	*Enterococcus faecium*	GPB	4.90E+04	ATCC 19434
CA0742	*Escherichia coli*	GNB	7.80E+04	ATCC 8739
CA0743	*Klebsiella pneumoniae*	GNB	1.80E+04	ATCC10031
CA0745	*Staphylococcus aureaus*	GPB	2.30E+05	ATCC 6538
CA0746	*Acinetobacter baumnnii*	GNB	2.70E+04	ATCC19606
CA0747	*Candida albicans*	Fungus	4.00E+02	ATCC 10231
CA0749	*Enterococcus faecalis*	GPB	1.80E+04	ATCC 51299
CA0750	*Staphylococcus epidermidis*	GPB	2.90E+04	ATCC 12228
CA0753	*Streptococcus pneumoniae*	GPB	1.00E+03	ATCC 49619
–	*Serratia marcescens*	GNB	1.00E+05	–
–	*Streptococcus agalactiae*	GPB	1.60E+04	–
–	*Streptococcus pyogenes*	GPB	2.20E+04	–

*: Nine of the standard strains were obtained from Gene Well Corp, Shenzhen, China, and three were isolated from the clinical laboratory. The method of Quantitative bacterial suspension was based on Flat colony counting method.

GPB: Gram positive bacteria; GNB: Gram negative bacteria

Supplemental Table 2. Clinical characteristics of enrolled patients.

	**SBP (n=66)**	**Bacterascites (n=37)**	**No-AFI (n=102)**	*P* ¶
Age (years, mean ± SD)	57 ± 9	61 ± 9*	59 ± 10	0.03
Gender (Male/Female)	57 / 9	34 / 3	78 / 24	0.06
Etiology, n (%)				
Alcohol	28（42.4）	21（56.8）	40 (39.2)	0.18
HBV	22 (33.3)	4 (10.8)	23 (22.5)	0.03
HCV	3 (4.5)	2 (5.4)	2 (2.0)	0.51
HBV plus alcohol	6 (9.1)	0 (0)	11 (10.8)	0.12
Others	7 (10.6)	10 (27.0)	26 (25.5)	0.04
Complications, n (%)				
Gastrointestinal bleeding	8 (12.1)	5 (13.5)	9 (8.9)	0.66
Hepatic encephalopathy	13 (19.7)	5 (13.5)	13 (12.7)	0.45
HRS	8 (12.1)	4 (10.8)	8 (7.8)	0.64
Antibiotic use, n (%)	54 (81.8)	21 (56.8)	38 (37.3)	0.02
Laboratory parameters				
WBC ($\times 10^9$/L), median (IQR)	6.1 (4.6,10.4)	3.7 (3.0,5.4)*	4.5（3.2, 7.0）*	<0.001
neutrophil (%), median (IQR)	80.3 (74.9,86.5)	65.4 (60.4,77.0)*	68.7 （60.2, 77.9）*	<0.001
ALT (IU/L), median (IQR)	18.0 (12.5,27.5)	15.0 (11.0,22.0)	18.0 (12.0,30.8)	0.26
AST(IU/L), median (IQR)	39.0 (20.0,67.5)	27.0 (20.0,44.0)	37.0 (24.3,65.8)	0.07
albumin (g/dL), mean ± SD	29.0 ± 4.4	29.3 ± 4.0	29.1 ± 4.0	0.99
Total bilirubin (μmol/L), median (IQR)	61.1 (30.3,181.9)	39.7 (21.1,52.8)*	46.1 (24.1,80.6)	<0.01
PTA, mean ± SD	58.7 ± 27.4	67.2 ± 20.0*	62.3 ± 19.0	0.04
Serum creatinine (μmol/L) , median (IQR)	84.0 (57,159.5)	81.0 (65.0,116.0)	75.0 (57.5,103.0)	0.66
Platelets (10^9/L), median (IQR)	84.0 (52.5,123.5)	58.0 (38.0,101.0)	80.5 (47.5,129.3)	0.17
Ascites WBC count ($\times 10^6$/L), median (IQR)	726 (356,2067)	229 (103,370)*	197 (99,308)*	<0.001

Ascites PMN count ($\times 10^6$/L), median (IQR)	420 (113,1276)	31 (15,58)*	25 (13,56)*	<0.001
Scores				
Child-Pugh	9.5 ± 2.6	8.8 ± 2.1	9.0 ± 2.5	0.16
MELD	13.6 ± 10.2	6.5 ± 7.5*	9.4 ± 8.1	0.01

SBP, spontaneous bacterial peritonitis; AFI, ascitic fluid infection; SD, standard deviation; IQR, interquartile range; HRS, hepatorenal syndrome; ALT, alanine aminotransferase; AST, aspartate aminotransferase; PTA, prothrombin activity; MELD, model for end-stage liver disease; PMN, polymorphonuclear neutrophils; WBC, white blood cell.

P¶ value from Kruskal-Wallis test for continuous variables or Fisher's exact test for discrete variables comparing patients with SBP to patients with bacterascites and No-AFI.

*$P < 0.05$ versus SBP

Supplemental Table 3. Qualitative detection of positive controls dilution replicates by probit analysis.

Name	LOD
Escherichia_coli	353.4
Enterococcus Faecium	114.3
Enterococcus faecalis	60.1
Staphylococcus epidermidis	828.2
Serratia marcescens	33.3
Streptococcus pyogenes	59.0
Klebsiella oxytoca	688.4
Staphylococcus aureus	42.7
Streptococcus agalactiae	42.8
Candida glabrata	579.4
Acinetobacter_baumannii	73.2
Klebsiella pneumoniae	46.9

LOD, limit of detection.

Supplemental Table 4. Interference results of mNGS testing.

proportion	Positive controls	nRPTM			CV value
		Replicate 1	Replicate 2	Replicate 3	
**1:10**	*Staphylococcus epidermidis*	38654	33478	35644	7.20%
	Staphylococcus aureus	377983	305116	344976	10.60%
**2:1**	*Enterococcus faecalis*	128727	113708	119995	6.20%
	Enterococcus Faecium	87375	80845	77661	6.00%

CV, Coefficient of Variance; nRPTM, normalized reads per ten million.

Supplemental Table 5. The clinical characteristics of 9 patients with positive mNGS detections and negative culture results.

Patients	Culture	mNGS	Digital PCR		Clinical adjudication
			G+	G-	
#1	Neg	*Enterococcus faecalis*	Pos	Neg	None
#2	Neg	*Enterococcus faecalis*	Pos	Neg	none
#3	Neg	*Candida Albicans*<br>*Schistosoma japonicum Katsurada*	Pos	Neg	PMN (938 cells/mm^3)
#4	Neg	*Enterococcus faecalis*	ND	ND	PMN (241 cells/mm^3), elevated PCT, abdominal pain and tenderness
#5	Neg	*Staphylococcus epidermidis*<br>*Candida parapsilosis*	Pos	Neg	none
#6	Neg	*Enterococcus faecalis*	ND	ND	PMN (27 cells/mm^3), gastrointestinal tract bleeding, abdominal tenderness, Child-Pugh grades C
#7	Neg	*Staphylococcus capitis*<br>*streptococcus mutans*<br>*Micrococcus luteus*	Neg	Neg	PMN (54 cells/mm^3)<br>hepatic encephalopathy, chronic liver failure
#8	Neg	*Staphylococcus patenkov*	Pos	Neg	None
#9	Neg	*Staphylococcus patenkov*	Pos	Neg	none

Neg, negative; Pos, positive; ND, not done; PMN, polymorphonuclear neutrophil.

Standard operating procedure.

Removal of host DNA

1) Place 10 μl internal control solution into 1ml sample, then vortex.

2) Add 500 μl NPB and 10 μl HLE, mixed by vertexing, and centrifuge briefly, respectively. Incubate at 1000 rpm for 15 min and then centrifuge at 12000 rpm for 10 min. Remove the supernatant.

3) Add 1 ml RS to resuspend the sediment, transfer the entire liquid to PBT, centrifuge at 12000 rpm for 5 min, discard 600 μl of supernatant.

DNA extraction

4) Add 100μl of DX, mixed by vertexing, and centrifuge briefly. Incubate at 56 °C, 1500 rpm for 10 min.

5) Transfer the supernatant to a new 2 mL tube and add 50 μl of PK, mixed by vertexing, and centrifuge briefly. Incubate at 56 °C, 1000 rpm for 10 min.

6) Add 400 μl of BLB, mixed by vertexing, and centrifuge briefly. Incubate at 56 °C, 1000 rpm for 10 min, and centrifuge briefly. Add 500 μl of isopropanol, mixed by vertexing, and centrifuge briefly.

7) Carefully transfer 700 μl of the solution to PSC (adsorption column into the collection tube) and centrifuge at 12,000 rpm for 1 min.

8) Replace with a new BCT2, transfer all the remaining liquid to PSC, centrifuge at 12000 rpm for 1 min. Replace with a new BCT2, add 500 μl PWB1, PWB2 for twice, centrifuge at 12000 rpm for 1 min, respectively. Centrifuge at 12,000 rpm for 3 min with a new BCT2. Transfer the PSCs to a new 1.5 mL tube and leave at room

temperature for 5 min.

9) Add 50 μl EB and leave at room temperature for 2 min and centrifuge at 12,000 rpm for 1 min. Transfer the eluate back into the PSC, leave it at room temperature for 2 min, centrifuge at 12,000 rpm for 1 min to get the final DNA solution.

Quantification

10) Take 197ul Qubit ds DNA Buffer and 1ul Reagent*200X concentrate in DMAO, and protect it from light after mixing.

11) Take 190ul dilution of Reagent*200X concentrate in DMAO, add10ul Qubit ds DNA HS Standard#1 and 10ul Qubit ds DNA HS Standard#2, 198ul solution mixed 2ul sample for 2 min.

12) Put the standard#1, standard#2 into the instrument for testing (standard#1 is the minimum value, standard#2 is the upper limit of the detection concentration of the kit), the sample to be tested is put into the instrument and the sample size of the sample is selected.

Tagmentation

Place AMPure XP Beads at room temperature for 30 min, then vortex violently. Place RSB (Resuspension Buffer) on the benchtop and thaw at room temperature.

13) Dissolve PEG8000 powder to RNase-free water with concentration of 50% (w/w), and filter the solution by 0.22 μm filter.

14) Combine the following volumes to prepare the tagmentation master mix. Prepare the tagmentation mix by combining the reagents listed below.

Component	volume
ATM (Nextera XT lib prep Kit)	5 μl
TB1 buffer	4 μl
50% PEG8000	2 μl

Total Volume	11 µl

15) Transfer 11 µl tagmentation master mix to each tube containing 9 µl sample rom step 5. Pipette each sample 10 times to resuspend. Incubate the reaction at 55°C for 15 min, then add 5 µl NT to each tube. Pipette to mix. Centrifuge at 280 × g at 20°C for 1 minute. Incubate at room temperature for 5 min.

Amplification

16) Add 5 µl each Index 1 (i7) adapter and 5 µl of each Index 2 (i5) adapter to each sample form step 8. Add 15 µl NPM to each tube containing index adapters. Pipette 10 times to mix. The volume is 50 µl.

17) Centrifuge at 280 × g at 20°C for 1 min. Place on the preprogrammed thermal cycler and run the PCR program as detailed below.

- ▶ 72℃ for 15 min
- ▶ 95℃ for 30 seconds
- ▶ 18 cycles of:
  - ▶ 95℃ for 10 seconds
  - ▶ 55℃ for 30 seconds
  - ▶ 72℃ for 30 seconds
- ▶ 72℃ for 5 min
- ▶ Hold at 10℃

18) For metagenomic sequencing, pool 8-16 libraries in equal volumes and go to **Step 21**.

Purification

This step uses AMPure XP beads to purify the library DNA and remove short library fragments.

19) Place AMPure XP Beads at room temperature for 30 min, then vortex violently. Place RSB （Resuspension Buffer）on the benchtop and thaw at room temperature.

20) Add 1.2×volume of beads to the PCR products and pipette 10 times to mix. Incubate the mixture at room temperature for 5 min. Then shake at 1800 rpm for 2 min, Incubate at room temperature for 5 min.

21) Place the tube on a magnetic stand and wait until the liquid is clear (~5 min).

22) Carefully remove and discard all supernatant from each well without disturbing beads.

23) Wash beads with 200µl 80% ethanol (freshly prepared) and incubate for 30s, then remove the ethanol. Repeat this step one more time. Using a 20 µl pipette, remove residual 80% EtOH from each tube.

24) Dry the beads on magnetic stand with cap open for 5min. Add 22 µl RSB (Resuspension Buffer) and close the cap, shake at 1800 rpm for 2 min, Incubate at room temperature for 5 min

25) Quickly spin down the tube then place it on magnetic stand until the solution is clear (~5 min).

26) Carefully aspirate 20µl supernatant to a clean tube without disturbing beads. The library can be restored at -20°C for 6 months.

Check Library Quality

27) Library quantitation. Run 1 µl of undiluted library on Qubit 2.0 Fluorometer using Qubit® dsDNA HS Assay Kits following the manufacturer's instructions.

28) Fragment length analysis. Run 1 µl of undiluted library on Agilent Technology 4200 Bioanalyzer using a High Sensitivity DNA chip or Agilent 4200 TapeStation using Agilent High Sensitivity DNA Kit following the manufacturer's instructions.
